# Standard knee radiographs enable deep learning inference of MRI-defined cartilage and meniscal damage in early knee osteoarthritis: a study using the osteoarthritis initiative database

**DOI:** 10.3389/fphys.2026.1858407

**Published:** 2026-06-10

**Authors:** Tariq Alkhatatbeh, Ahmad Alkhatatbeh, Hao Liao, Yan Liao, Zhilin Zhang, Hang Fang, Weidong Chen, Dongfeng Wu, Rongkai Zhang

**Affiliations:** 1 Department of Joint Surgery, Center for Orthopaedic Surgery, The Third Affiliated Hospital of Southern Medical University, Academy of Orthopedics, Guangzhou, Guangdong, China; 2 Orthopedic Hospital of Guangdong Province, Guangzhou, Guangdong, China; 3 Guangdong Provincial Key Laboratory of Bone and Joint Degeneration Diseases, Guangzhou, Guangdong, China; 4 Department of Orthopedics, The First Affiliated Hospital of Shantou University Medical College, Shantou, Guangdong, China; 5 Department of Information Management, The Third Affiliated Hospital of Southern Medical University, Academy of Orthopedics, Guangzhou, Guangdong, China

**Keywords:** cartilage damage, deep learning, knee osteoarthritis, meniscal damage, radiography

## Abstract

**Background:**

To develop and validate a radiograph-only deep learning framework that jointly predicts magnetic resonance imaging (MRI)-defined early structural pathology in knees with early radiographic osteoarthritis (Kellgren-Lawrence [KL] grades 0-1) and performs full-spectrum KL classification across all grades within a single multi-task architecture.

**Methods:**

This retrospective study used baseline data from the Osteoarthritis Initiative (OAI) comprising 8260 knees (4130 participants). A multi-task ConvNeXt-Base network was trained with five-fold stratified group cross-validation enforcing strict subject-level data separation. KL classification was supervised across all knees. MRI prediction heads were activated only for KL 0–1 knees with available labels. MRI Osteoarthritis Knee Score (MOAKS)-derived binary endpoints were defined at a threshold of ≥2: tibiofemoral cartilage damage (primary endpoint) and meniscal morphology damage (secondary endpoint).

**Results:**

KL grading performance across held-out folds was as follows: mean quadratic weighted kappa (QWK) 0.8284 (standard deviation [SD] 0.0255), balanced accuracy 0.6836 (SD 0.0159). In KL0/1 knees with both MRI labels (n = 2561), prevalence was 31.3% for cartilage damage and 26.4% for meniscal damage. Mean AUROC/AUPRC were 0.7329 (SD 0.0203)/0.5741 (SD 0.0364) for cartilage damage and 0.7193 (SD 0.0533)/0.5147 (SD 0.0719) for meniscal damage.

**Conclusions:**

A leakage-controlled multi-task radiograph model achieved strong KL grading agreement and moderate discrimination of MRI-defined cartilage and meniscal pathology in knees with early radiographic osteoarthritis, supporting a potential role as an assistive triage signal to identify patients who may benefit from earlier MRI evaluation. However, clinical correlation remains essential; MRI referral decisions should always be made in the context of the individual patient’s symptoms, functional impairment, and overall clinical assessment, and cannot be based on AI-derived probability scores alone.

## 1 Introduction

Knee osteoarthritis (KOA) is one of the most prevalent musculoskeletal disorders worldwide and a leading cause of chronic pain and functional disability in adults (Sharma, 2021). In clinical practice, the plain anteroposterior knee radiograph remains the primary imaging investigation for initial assessment, affording a practical and cost-effective means of structural staging using the Kellgren-Lawrence (KL) classification system (Roemer et al., 2020; Roemer et al., 2022). While radiographs reliably demonstrate joint space narrowing, osteophyte formation, and subchondral changes associated with established disease, they are intrinsically insensitive to soft tissue structures. Articular cartilage and the menisci are not directly visible on conventional radiographs, and meaningful structural damage to these tissues can be present well before radiographic hallmarks become apparent, particularly in knees classified as KL grade 0 or 1 (Roemer et al., 2020; Roemer et al., 2022).

The clinical relevance of this discordance between radiographic and tissue-level disease has been underscored by magnetic resonance imaging (MRI) studies in large population cohorts. Chang et al. recently demonstrated that MRI-defined structural osteoarthritis rarely occurs in the absence of cartilage damage, and that knees satisfying MRI-based osteoarthritis definitions are associated with clinically significant progression over extended follow-up (Chang et al., 2025; Chang et al., 2026). These data indicate that a non-trivial proportion of patients presenting with radiographically mild or doubtful findings may already harbor definite cartilage and meniscal structural abnormalities, creating a window for earlier clinical decision-making that is currently missed when management is guided by radiographs alone.

Artificial intelligence (AI) applied to musculoskeletal radiology has advanced rapidly over the past decade, and deep learning models for knee radiograph analysis have achieved high agreement with expert radiologists for KL grading tasks (Tiulpin and Saarakkala, 2020; Swiecicki et al., 2021; Kijowski et al., 2023; Brejnebøl et al., 2024; Zhao et al., 2024; Vaattovaara et al., 2025). A 2025 meta-analysis confirmed strong discriminative performance across architectures, though classification of the earliest grades (KL 0 versus 1) remains comparatively challenging (Zhao et al., 2024). In parallel, deep learning applied directly to MRI has demonstrated excellent diagnostic accuracy for meniscal tears and cartilage lesions, with reported area under the curve (AUC) values typically exceeding 0.90 in prospective and multi-center studies (Roblot et al., 2019; Fritz et al., 2020; Rizk et al., 2021; Güngör et al., 2025). However, these MRI-input models require MRI acquisition at the point of assessment and are not applicable to the large proportion of patients who initially undergo radiographic evaluation only.

The present study addresses a clinically distinct and relatively unexplored question: whether standard posteroanterior fixed-flexion knee radiographs can serve as a source of inferential signal for MRI-defined soft tissue pathology in knees with early radiographic osteoarthritis, while simultaneously retaining full-range KL classification. To our knowledge, a radiograph-only deep learning approach combining full-spectrum KL grading with early-KL-supervised MRI Osteoarthritis Knee Score (MOAKS)-based cartilage and meniscal endpoint prediction has not been systematically reported. We hypothesized that shared convolutional feature learning from the full radiograph cohort through the KL grading task would enable development of backbone representations informative for MRI-defined soft tissue pathology in the early-disease subgroup, achieving discrimination above uninformative baseline. The primary aim was prediction of MRI-defined tibiofemoral cartilage damage (MOAKS score ≥2) in knees with early radiographic osteoarthritis (KL grades 0–1). The secondary aim was prediction of MRI-defined meniscal morphology damage (MOAKS score ≥2) in the same subgroup, alongside full-spectrum KL classification as a co-primary structural output.

## 2 Material and methods

### 2.1 Study design and data source

This was a retrospective computational modeling study using baseline cross-sectional data from the Osteoarthritis Initiative (OAI), a publicly available, multicenter, longitudinal observational cohort of adults aged 45–79 years with or at risk for symptomatic KOA. The analytic cohort comprised 8260 knees from 4130 participants (one left and one right knee per participant). The study constitutes a secondary analysis of de-identified data and was conducted in accordance with the ethical standards of the OAI’s originating institutional review boards. KL grade labels derived from posteroanterior fixed-flexion radiographs were available from OAI source files for all 8260 knees and covered the full grade spectrum (KL0 to KL4). The observed KL distribution was: KL0, n = 3253; KL1, n = 1495; KL2, n = 2175; KL3, n = 1086; and KL4, n = 251.

**Figure 1:**
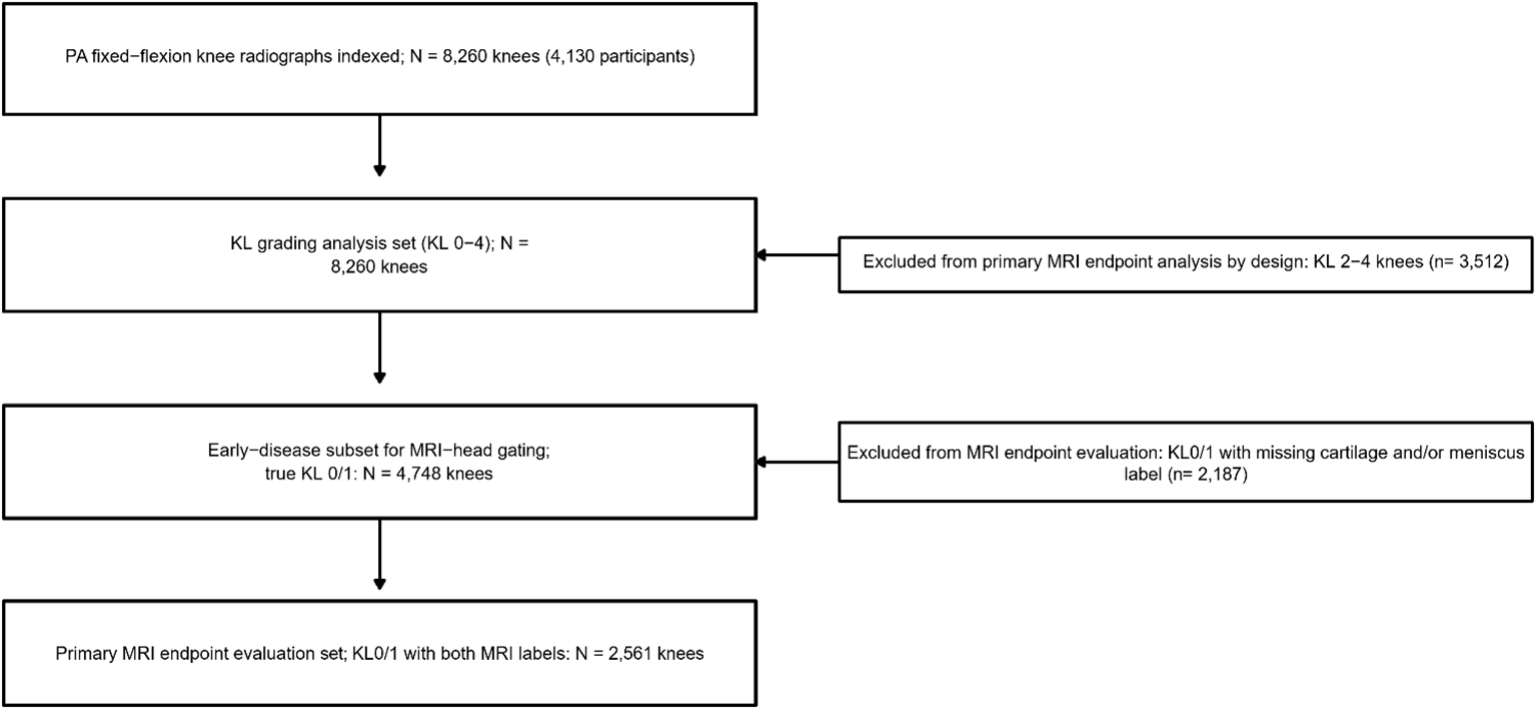
Study flow and analysis sets. Flow diagram of cohort derivation from baseline osteoarthritis initiative radiographs; the full analytic cohort included 8,260 knees and 4,748 knees were KL0/1; the primary MRI-endpoint analysis set included 2,561 KL0/1 knees with both MRI-derived labels available, while 2,187 KL0/1 knees were excluded from MRI-endpoint evaluation because one or both MRI labels were missing.

**Figure 2:**
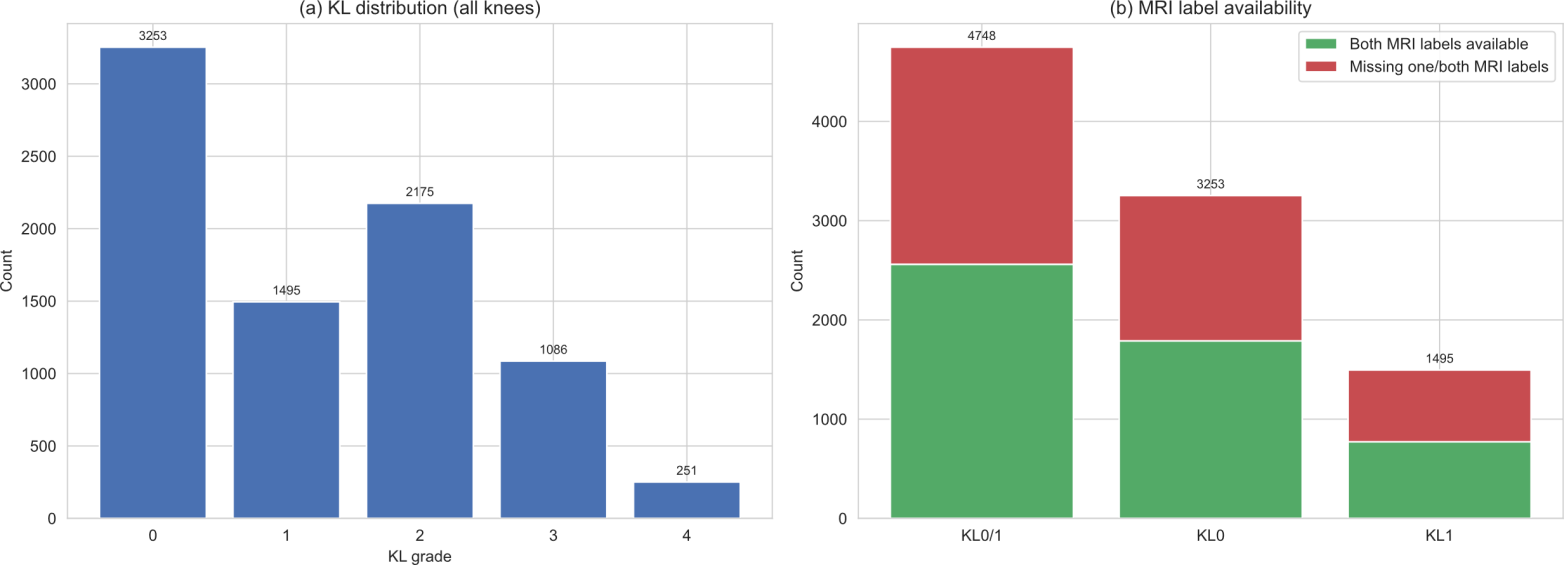
Cohort composition. **(a)** Distribution of Kellgren-Lawrence (KL) grades in the full cohort; **(b)** MRI label availability for the primary endpoints (tibiofemoral cartilage damage and meniscal morphology damage) in KL0/1, KL0, and KL1 strata; bars show knees with both labels available versus missing one or both labels.

MRI endpoint labels were derived from baseline MRI Osteoarthritis Knee Score (MOAKS) clinical data files available through the OAI (Lester, 2016). Across all KL grades, 3673 knees had at least one MRI endpoint label available. Because MRI supervision was restricted by design to early radiographic disease, the primary MRI evaluation cohort was defined as KL0/1 knees with complete labels for both MRI endpoints (n = 2561 of 4748 KL0/1 knees). The study flow, including all inclusion and exclusion steps, is summarized in **Figure 1** and the counts per class are shown in **Figure 2**.

### 2.2 MRI endpoint construction

MRI targets were derived from MOAKS baseline variables by region-wise aggregation to knee-level binary outcomes. MOAKS scores were parsed from the numeric prefix of each variable string. The primary endpoint used tibiofemoral cartilage morphology codes from femoral central and posterior subregions and tibial subregions, specifically excluding patellofemoral regions, given the distinct biomechanical role and clinical relevance of the tibiofemoral compartment in early OA. The secondary endpoint used meniscal morphology codes from medial and lateral meniscal regions across their anterior, body, and posterior zones.

For each endpoint, the maximum regional MOAKS score per knee was computed, and a knee was classified as positive if this maximum score was ≥2. In the MOAKS framework, grade 2 reflects at least moderate structural abnormality, whereas grade 1 may capture milder findings; therefore, ≥2 was pre-specified to target clinically meaningful damage for triage-focused modeling. This thresholding approach is consistent with semiquantitative MOAKS interpretation and prior OAI-oriented structural analyses (Lester, 2016; Astuto et al., 2021). Knee-level positivity reflected presence of definite structural damage without spatial localization; detailed MOAKS variable codes and decision rules are provided in **Table 1**.

**Table 1:** MRI endpoint definitions derived from MOAKS variables and their clinical interpretation.

Endpoint	MOAKS regions (OAI variable codes)	Knee-level decision rule	Clinical interpretation
MRI-defined tibiofemoral cartilage damage (primary)	Femoral central/posterior and tibial subregions (V00MCMFMC, V00MCMFLC, V00MCMFMP, V00MCMFLP, V00MCMTMA, V00MCMTLA, V00MCMTMC, V00MCMTLC, V00MCMTMP, V00MCMTLP); patellofemoral regions excluded	Positive if maximum regional cartilage morphology score ≥2	Definite tibiofemoral cartilage structural damage at knee level (non-localizing composite)
MRI-defined meniscal morphology damage (secondary)	Medial and lateral meniscal morphology regions (V00MMTMA, V00MMTMB, V00MMTMP, V00MMTLA, V00MMTLB, V00MMTLP)	Positive if maximum regional meniscal morphology score ≥2	Definite meniscal structural damage at knee level (non-localizing composite)

### 2.3 Model architecture and training strategy

**Figure 3:**
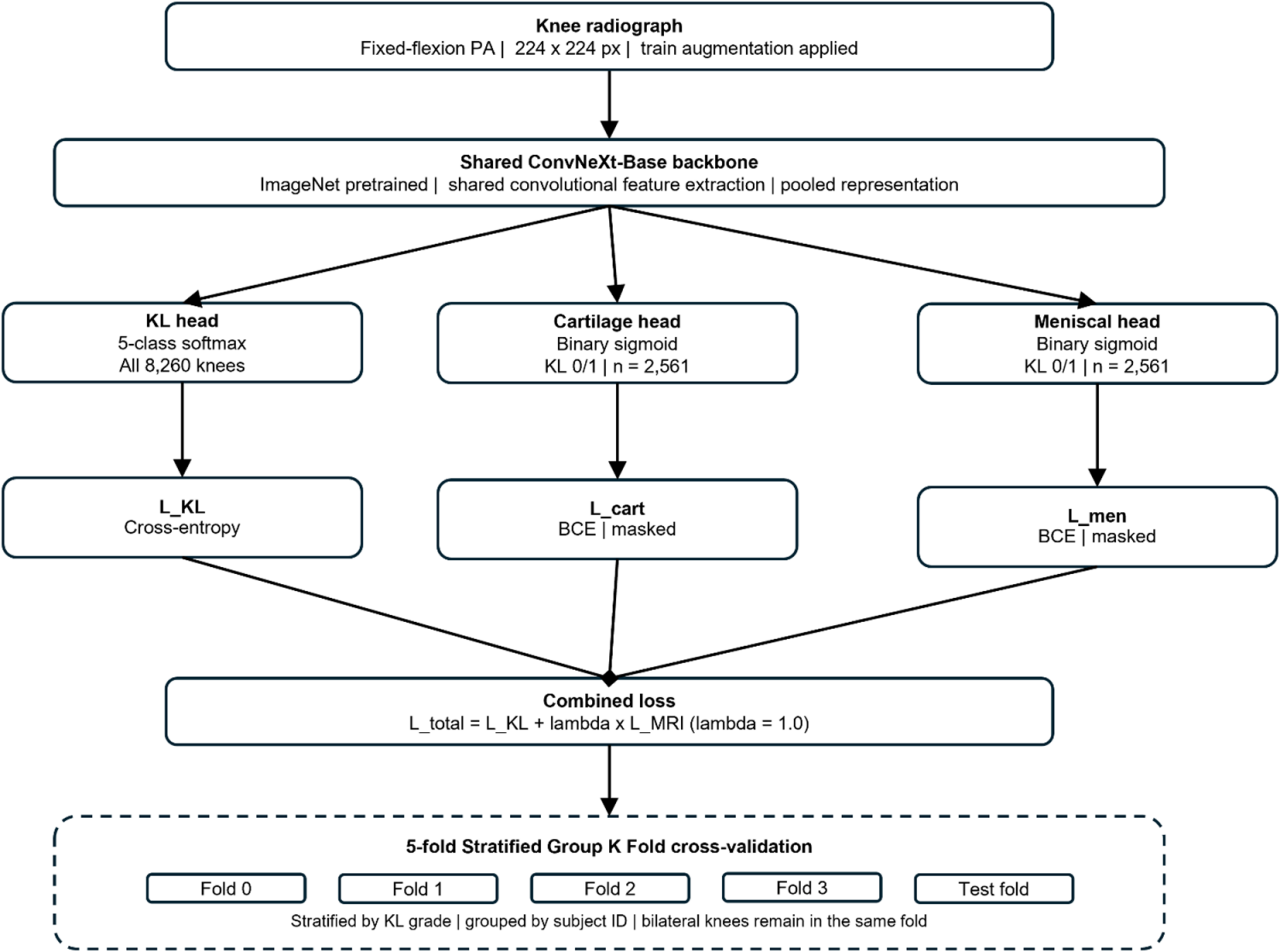
Model architecture and training pipeline. Posteroanterior fixed-flexion knee radiographs are preprocessed and passed through a shared ConvNeXt-base backbone. Three task-specific heads branch from the pooled feature representation: a five-class softmax head for full-spectrum KL grading (all 8,260 knees) and two independent sigmoid heads for MRI-defined tibiofemoral cartilage damage and meniscal morphology damage (KL0/1 gated; n = 2,561). MRI gradients gated by ground-truth KL grade and label availability. Training employed five-fold StratifiedGroupKFold cross-validation with stratification by KL grade and subject-level grouping to prevent data leakage. KL Kellgren–Lawrence; MOAKS MRI osteoarthritis knee score; BCE binary cross-entropy.

A ConvNeXt-Base backbone initialized with ImageNet-pretrained weights was adopted as the feature extractor, given its demonstrated superiority over canonical convolutional architectures on image classification benchmarks and its strong transfer learning characteristics for medical imaging tasks. Three output heads were attached: a five-class softmax head for KL classification (grades 0–4) and two independent sigmoid heads for binary MRI endpoint prediction (tibiofemoral cartilage damage and meniscal morphology damage) (**Figure 3**).

The overall training objective combined KL classification loss with masked MRI binary cross-entropy losses. The KL head received supervision from all 8260 knees in each training fold. Each MRI head was activated only when the ground-truth KL grade was 0 or 1 and the corresponding MRI label was available; no MRI gradient was propagated for knees with KL ≥2 or missing MRI labels. The total loss was:


$$L_{total}=\;L_{KL}+\;\lambda \;\times \;L_{MRI}.$$

Where *L_total* is the total optimization objective per mini-batch; *L_KL* is the KL-grade classification loss (multiclass cross-entropy over KL classes 0-4) computed for all samples; *L_MRI* is the masked MRI multi-task loss (mean binary cross-entropy across the two MRI endpoints) computed only for samples with true KL in {0,1} and available MRI labels; and *lambda* is the scalar weighting coefficient controlling the contribution of L_MRI relative to *L_KL* (baseline: *lambda* = 1.0). This gating design ensured that the soft tissue inference capacity of the model was developed exclusively from the early-disease subgroup, reflecting the intended clinical deployment scenario.

Images were converted to three-channel RGB, resized to 224 × 224 pixels, and normalized using ImageNet channel statistics [mean (0.485, 0.456, 0.406), standard deviation (0.229, 0.224, 0.225)]. Training-phase augmentation comprised random horizontal flipping (probability 0.5) and random rotation (± 7°), with no geometric augmentation at validation/test time. Optimization used AdamW with initial learning rate 3 × 10^-4^ and weight decay 1 × 10^-4^, batch size 64, mixed-precision training, and maximum 20 epochs with early-stopping patience of 7 epochs. No learning-rate scheduler, focal-loss reweighting, or gradient clipping was used in the primary model. Hyperparameters were fixed *a priori* and held constant across all folds (no fold-specific optimization). To support reproducibility, random seeds were fixed for Python, NumPy, and PyTorch, deterministic CUDA settings were enabled, and each fold archived a run manifest (configuration snapshot, environment metadata, and package list). Full configuration details are given in **Table 2**.

**Table 2:** Model architecture, training configuration, and reproducibility settings.

Parameter	Configuration
Backbone	ConvNeXt-Base (convnext_base.fb_in22k_ft_in1k), ImageNet pretrained
Input	RGB radiographs, resized to 224 × 224 pixels
Training augmentation	Random horizontal flip (*p* = 0.5), random rotation (± 7°), ImageNet normalization
Validation/test augmentation	Deterministic resize and ImageNet normalization only
Output heads	KL head: 5-class softmax (grades 0–4); MRI heads: two independent sigmoid outputs (cartilage, meniscus)
Loss function	L_total = L_KL + λ × L_MRI; KL head: multi-class cross-entropy; MRI heads: binary cross-entropy with logits; λ = 1.0
MRI loss gating	Active only when true KL ∈ {0, 1} and label is present; no MRI supervision for KL ≥2 or missing labels
Optimizer	AdamW
Learning rate/weight decay	3 × 10^-4^/1 × 10^-4^
Batch size/gradient accumulation	64/1
Max epochs/early stopping patience	20/7 epochs without validation improvement
Mixed precision	Automatic mixed precision (AMP) with gradient scaling, CUDA
Cross-validation	5-fold StratifiedGroupKFold; stratified by KL grade; grouped by subject identifier
Checkpoint selection	Primary: highest validation QWK; tie-breaker: mean validation AUPRC across both MRI endpoints in KL0/1
Hardware	Single workstation: NVIDIA GeForce RTX 5080 (16 GB VRAM), Intel CPU, 48 GB RAM
Seed/reproducibility	Fixed random seed; config snapshots; requirements logged; TensorBoard and CSV metric logging

### 2.4 Cross-validation, leakage prevention, and checkpoint selection

Five-fold stratified group cross-validation was implemented using StratifiedGroupKFold with stratification by KL grade and grouping by subject identifier, ensuring that both knees from the same participant were assigned exclusively to a single fold. This subject-level constraint strictly prevented patient-level data leakage between training and held-out evaluation partitions. Fold sizes were balanced at approximately 1650–1654 knees per fold; held-out KL0/1 knees with complete MRI labels across folds were 494, 506, 532, 505, and 524, respectively.

Checkpoint selection followed a pre-specified, fixed hierarchy: the checkpoint with the highest validation QWK for KL classification was selected as the primary criterion, with mean validation AUPRC across both MRI endpoints in KL0/1 serving as tie-breaker. This selection policy was locked before final cross-validation evaluation to prevent *post-hoc* tuning.

### 2.5 Statistical analysis and performance metrics

KL grading performance was assessed in all held-out knees per fold using QWK (primary KL metric), overall accuracy, balanced accuracy, and macro-averaged F1 score. MRI endpoint performance was evaluated in held-out KL0/1 knees with complete MRI labels using AUROC, AUPRC, and Brier score. Threshold-dependent operating points were assessed at a fixed threshold of 0.5 and at a best-F1 threshold derived on the validation fold and applied to the corresponding held-out fold. Cross-validation summaries are reported as mean and standard deviation across folds with t-based 95% confidence intervals. For MRI endpoint discrimination, we additionally computed pooled out-of-fold percentile bootstrap 95% confidence intervals (4,000 resamples).

A pre-specified sensitivity analysis evaluated MRI endpoint discrimination when the evaluation gate was set by predicted KL (predicted KL *∈* {0, 1}) rather than true KL, quantifying expected cascade loss in fully automated deployment. Differences between true-KL and predicted-KL gating were assessed using exact paired sign-flip permutation tests on fold-level AUPRC differences.

### 2.6 Uncertainty quantification (*post hoc*)

As a *post-hoc* uncertainty analysis, we applied class-conditional (Mondrian) split-conformal prediction to out-of-fold probabilities. For each test fold, conformal thresholds were calibrated on the remaining four folds using nonconformity score 1 − *p_y*, where *p_y* is the predicted probability assigned to class *y*. We report empirical coverage and prediction-set size (singleton vs multi-label sets) at nominal error rates *α* = 0.10 and *α* = 0.20, and calibration reliability using 10-bin expected calibration error.

### 2.7 Ablation analysis: multi-task versus single-task baselines

To evaluate the contribution of shared backbone training to MRI endpoint performance, we trained two single-task baseline models using the identical ConvNeXt-Base backbone, hyperparameters, augmentation pipeline, and cross-validation splits as the multi-task framework. Each single-task model was trained exclusively on the KL0/1 MRI-labeled subset (n = 2,561), predicting one MRI endpoint only, and thus did not receive any KL supervision from the full 8,260-knee cohort. This comparison directly isolates the representation-learning advantage attributable to the multi-task design, where the KL supervision signal provides the backbone with richer feature learning from a substantially larger training pool. Identical AUROC and AUPRC metrics were computed for comparison.

## 3 Results

### 3.1 Cohort characteristics and MRI endpoint prevalence

The analytic cohort comprised 8260 knees from 4130 participants, of which 4748 knees were classified as KL grade 0 or 1. Among KL0/1 knees, 2561 (53.9%) had complete labels for both MRI endpoints and constituted the primary MRI evaluation cohort; the remaining 2187 KL0/1 knees were excluded from MRI endpoint evaluation solely due to missing labels. The cohort composition and per-stratum label availability are detailed in **Table 3**.

**Table 3:** Cohort composition and MRI endpoint label availability by Kellgren-Lawrence grade.

Stratum	Total knees, n	Knees with both MRI labels, n	Label availability, %
All knees	8260	3673	44.5
KL grade 0	3253	1789	55.0
KL grade 1	1495	772	51.6
KL grade 2	2175	570	26.2
KL grade 3	1086	404	37.2
KL grade 4	251	138	54.9
KL 0–1 combined	4748	2561	53.9

To assess potential selection bias from incomplete MRI labels, we compared KL0/1 knees included in MRI-endpoint evaluation (both labels present; n = 2,561) versus excluded knees (missing one or both labels; n = 2,187). Label completeness was similar across KL strata: 55.0% in KL0 (1,789/3,253) and 51.6% in KL1 (772/1,495), corresponding to a small standardized mean difference for KL1 proportion (SMD = -0.063). Age differences were also small (included: 59.50 ± 8.96 years; excluded: 60.15 ± 9.35 years; SMD = -0.071) (**Supplementary Table 1**).

Within the KL0/1 labeled subset (n = 2561), the prevalence of MRI-defined tibiofemoral cartilage damage was 31.3% (801/2561 knees), increasing from 21.6% in KL0 knees (386/1789) to 53.8% in KL1 knees (415/772). MRI-defined meniscal morphology damage was present in 26.4% of the labeled subset (675/2561 knees), rising from 20.4% in KL0 (365/1789) to 40.2% in KL1 (310/772).

For endpoint-definition sensitivity, prevalence across alternative MOAKS cutoffs in the KL0/1 labeled cohort was: cartilage 55.5% at ≥1, 31.3% at ≥2, and 0.7% at ≥3; meniscal morphology 53.8% at ≥1, 26.4% at ≥2, and 20.9% at ≥3. These distributions support ≥2 as a pragmatic compromise between overinclusive mild-positive labeling and excessive sparsity for stricter cartilage thresholds.

### 3.2 Model training and convergence

Across the 5-fold cross-validation, the multi-task ConvNeXt model showed stable optimization behavior with the predefined stopping policy (maximum 20 epochs; early-stopping patience 7). Training terminated at epochs 17, 17, 19, 20, and 20 for folds 0-4, respectively, and the selected best checkpoints occurred at epochs 10, 10, 12, 20, and 13 (mean best epoch, 13.0; SD, 4.1). Checkpoint selection followed the prespecified hierarchy (primary: validation QWK; tie-break: validation mean MRI AUPRC in KL0/1).

**Figure 4:**
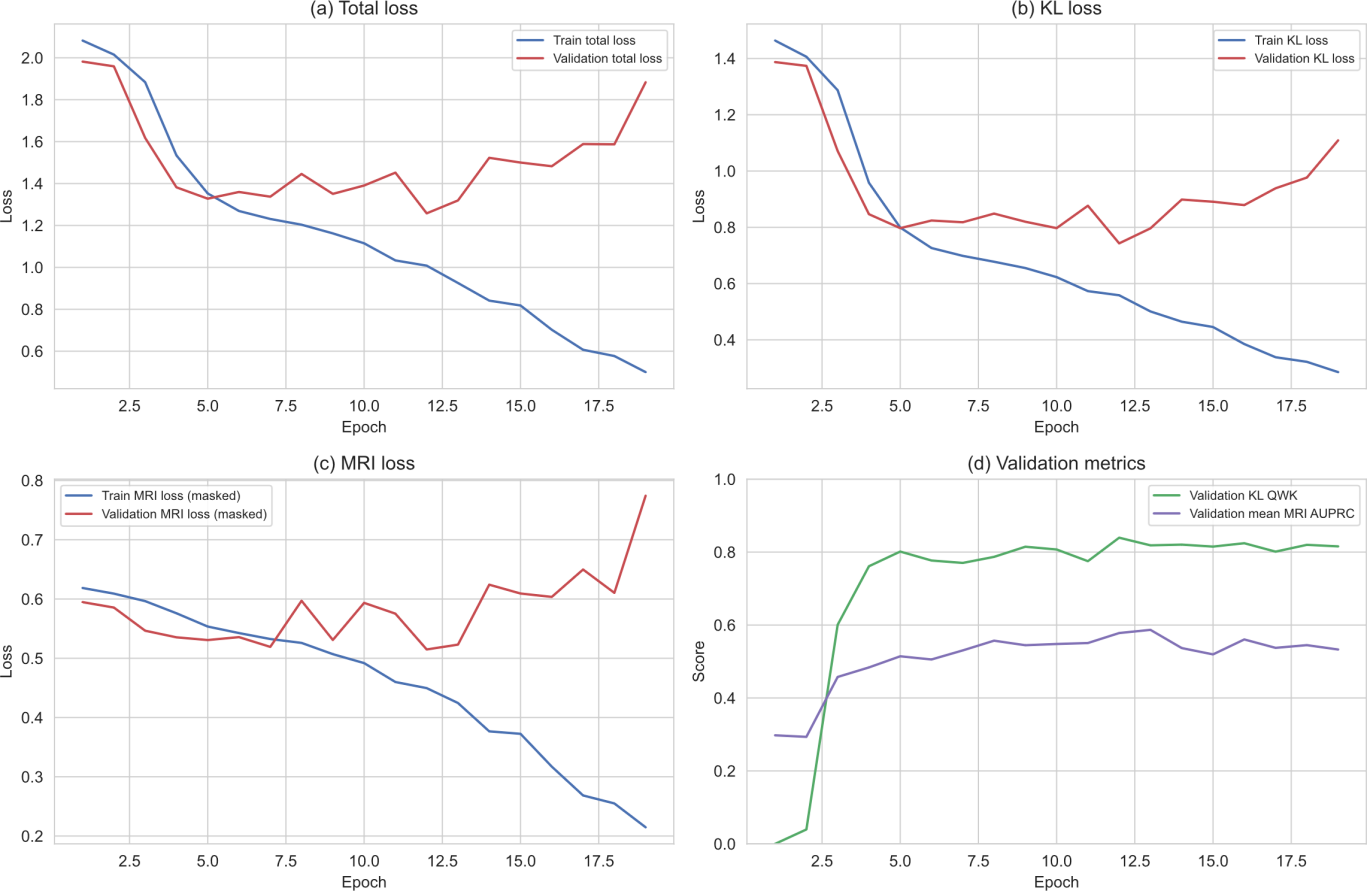
Training dynamics (representative median-QWK fold). **(a)** Train/validation total loss by epoch; **(b)** Train/validation KL loss by epoch; **(c)** Train/validation masked MRI loss by epoch; **(d)** Validation KL QWK and validation mean MRI AUPRC by epoch.

Training dynamics are shown in **Figure 4**. Train and validation total loss, KL loss, and masked MRI loss generally decreased in parallel (**Figures 4A-C**), without marked train-validation divergence suggestive of severe overfitting. Validation QWK and validation mean MRI AUPRC increased early and then plateaued (**Figure 4D**), consistent with convergence. Fold 3 showed slower optimization and a higher validation-loss floor than the other folds, which was concordant with its lower downstream performance. Full fold-wise learning trajectories are provided in **Supplementary Figure 3**.

### 3.3 KL grading performance

**Figure 5:**
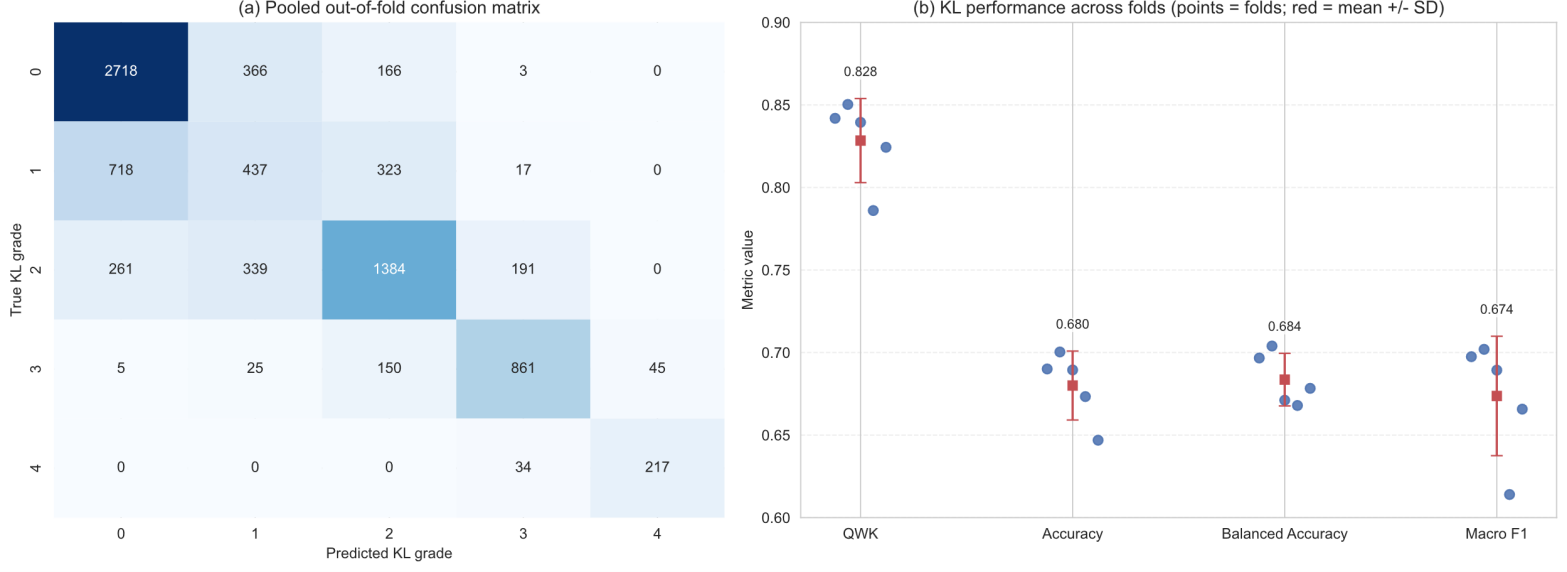
KL test-set performance summary (two-panel image) **(a)** Pooled out-of-fold confusion matrix from the held-out test partition of each fold (5-fold cross-validation) for KL grades 0-4, where rows indicate true KL grade and columns indicate predicted KL grade; **(b)** Fold-wise KL performance (QWK, accuracy, balanced accuracy, macro-F1) shown as points for each fold with mean +/- SD overlays.

KL grading performance across five held-out cross-validation folds showed mean QWK of 0.8284 (SD 0.0255; approximate 95% CI 0.797-0.860). Mean overall accuracy was 0.6800 (SD 0.0209; 95% CI 0.654-0.706), mean balanced accuracy was 0.6836 (SD 0.0159; 95% CI 0.664-0.703), and mean macro F1 score was 0.6737 (SD 0.0362; 95% CI 0.629-0.719). Per-fold QWK values were 0.8419, 0.8503, 0.8394, 0.7860, and 0.8243, indicating low inter-fold variability. Error analysis demonstrated that 94.2% of all out-of-fold predictions fell within ±1 grade of the true KL label, consistent with near-neighbor grade confusion patterns typical of ordinal classification (**Figure 5**).

### 3.4 MRI endpoint discrimination in KL 0–1 knees

**Figure 6:**
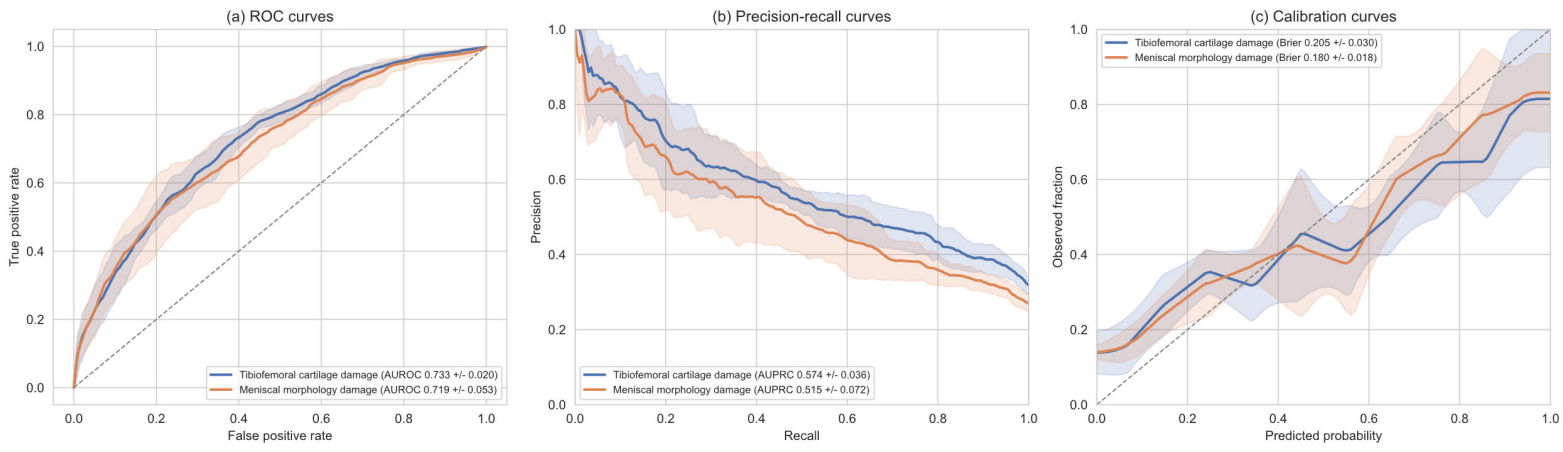
MRI endpoint performance in KL0/1 knees (5-fold mean +/- SD). **(a)** Receiver operating characteristic (ROC) curves for MRI-defined tibiofemoral cartilage damage and MRI-defined meniscal morphology damage; **(b)** Precision-recall (PR) curves for the same endpoints; **(c)** Calibration curves with perfect-calibration diagonal; shaded regions represent +/-1 SD across folds.

As shown in **Figure 6**, the primary endpoint of MRI-defined tibiofemoral cartilage damage, mean AUROC was 0.7329 (SD 0.0203; 95% CI 0.708-0.758) and mean AUPRC was 0.5741 (SD 0.0364; 95% CI 0.529-0.619). Mean Brier score was 0.2047 (SD 0.0296). At the fixed threshold of 0.5, mean sensitivity was 0.435 and mean specificity was 0.842. Using the validation-derived best-F1 threshold (mean 0.185; range 0.037-0.343 across folds), sensitivity increased to 0.739 with corresponding specificity of 0.627.

For the secondary endpoint of MRI-defined meniscal morphology damage, mean AUROC was 0.7193 (SD 0.0533; 95% CI 0.653-0.785) and mean AUPRC was 0.5147 (SD 0.0719; 95% CI 0.426-0.604). Mean Brier score was 0.1797 (SD 0.0183) (**Figure 6**). At threshold 0.5, mean sensitivity was 0.301 and specificity was 0.919, indicating a conservative operating point with high specificity at default threshold. At the best-F1 threshold (mean 0.164; range 0.027-0.276), sensitivity rose to 0.683 with specificity 0.652. Mean MRI AUROC across both endpoints was 0.7261 and mean AUPRC was 0.5444 (SD 0.0478). Full performance data are presented in **Table 4**, and fold-level operating-point summaries are provided in **Supplementary Table 6**. Per-fold ROC/PR curves and calibration plots are shown in **Supplementary Figure 1** and **Supplementary Figure 2**.

**Table 4:** Model performance across five held-out cross-validation folds.

Task/subgroup	Metric	Mean (SD)	Approx. 95% CI	Paired value
KL grading (all knees, n = 8260)	QWK	0.8284 (0.0255)	0.797–0.860	
	Accuracy	0.6800 (0.0209)	0.654–0.706	
	Balanced accuracy	0.6836 (0.0159)	0.664–0.703	
	Macro F1 score	0.6737 (0.0362)	0.629–0.719	
MRI-defined cartilage damage (KL0/1, n = 2561; prevalence 31.3%)	AUROC	0.7329 (0.0203)	0.708–0.758	
	AUPRC	0.5741 (0.0364)	0.529–0.619	
	Brier score	0.2047 (0.0296)		
	Sensitivity (threshold 0.5)	0.435		0.842 (specificity)
	Sensitivity (best-F1 threshold)	0.739		0.627 (specificity)
MRI-defined meniscal damage (KL0/1, n = 2561; prevalence 26.4%)	AUROC	0.7193 (0.0533)	0.653–0.785	
	AUPRC	0.5147 (0.0719)	0.426–0.604	
	Brier score	0.1797 (0.0183)		
	Sensitivity (threshold 0.5)	0.301		0.919 (specificity)
	Sensitivity (best-F1 threshold)	0.683		0.652 (specificity)

For clinical context, AUPRC should be interpreted relative to the no-skill baseline equal to the positive-class prevalence. For tibiofemoral cartilage damage (prevalence 31.3%), the no-skill AUPRC is 0.313; the observed mean AUPRC of 0.574 represents approximately 1.83-fold enrichment over uninformative random prediction. For meniscal morphology damage (prevalence 26.4%), the no-skill AUPRC is 0.264; the observed mean AUPRC of 0.515 represents approximately 1.95-fold enrichment. Regarding the Brier score, the prevalence-based reference value is p(1−p): 0.215 for the cartilage endpoint and 0.194 for the meniscal endpoint. The observed mean Brier scores (0.205 and 0.180, respectively) indicate marginal improvement in calibration above the prevalence-only baseline.

### 3.5 Subgroup analyses (KL stratum and age)

In subgroup analyses using out-of-fold predictions from the same 5-fold model, AUROC/AUPRC for cartilage damage were 0.701/0.403 in KL0 and 0.679/0.729 in KL1; for meniscal damage they were 0.693/0.412 in KL0 and 0.697/0.628 in KL1. Age-stratified analyses (<55, 55-64, and ≥ 65 years) showed moderate AUROC across strata, with AUPRC increasing in strata with higher endpoint prevalence (notably KL1 and older age groups) (**Supplementary Table 4**).

### 3.6 Sensitivity analysis: predicted-KL gating

When evaluation was gated by predicted rather than true KL grade (restricting to knees with predicted KL ∈ {0,1}), mean AUPRC across both MRI endpoints decreased from 0.5444 to 0.5123 (absolute difference -0.0321). Endpoint-level AUPRC changes were -0.0338 for cartilage damage and -0.0303 for meniscal morphology damage. In paired fold-wise testing, the mean AUPRC difference was not statistically significant (exact two-sided sign-flip *p* = 0.375), indicating directional but uncertainty-bounded cascade loss from upstream KL prediction errors (**Supplementary Figure 4**; **Supplementary Table 2**).

### 3.7 Uncertainty quantification results

Conformal uncertainty analysis showed near-nominal empirical coverage for both endpoints. For cartilage, empirical coverage was 0.900 at α = 0.10 and 0.800 at α = 0.20, with singleton prediction rates of 0.380 and 0.670, respectively. For meniscal damage, empirical coverage was 0.892 at α = 0.10 and 0.796 at α = 0.20, with singleton rates of 0.350 and 0.652. Pooled out-of-fold expected calibration error was 0.122 for cartilage and 0.091 for meniscal predictions (**Supplementary Table 3**).

### 3.8 Ablation analysis: multi-task versus single-task comparison

Single-task ConvNeXt-Base baselines trained exclusively on the KL0/1 labeled subset (n = 2,561) without KL supervision from the broader cohort achieved mean AUROC/AUPRC of 0.6617/0.4683 for tibiofemoral cartilage damage and 0.6247/0.3668 for meniscal morphology damage, with overall mean AUPRC of 0.4175. Compared with the multi-task model, the multi-task design yielded absolute AUROC improvements of +0.071 for cartilage and +0.095 for meniscal endpoints, and absolute AUPRC improvements of +0.106 and +0.148, respectively. Overall mean AUPRC improved by +0.127 (from 0.4175 to 0.5444). These consistent and substantial improvements across both endpoints and both metrics confirm that shared backbone training from the full 8,260-knee KL cohort provides a meaningful representation-learning advantage for the data-sparse MRI inference tasks, validating the multi-task design hypothesis (**Table 5**). Exploratory age+KL fusion analyses are summarized in **Supplementary Table 5**.

**Table 5:** Ablation analysis: multi-task versus single-task model performance for MRI endpoints in KL0/1 knees (5-fold means).

Category	Metric	Model A	Model B	Δ (improvement)
Cartilage	AUROC	0.7329	0.6617	+0.071
Cartilage	AUPRC	0.5741	0.4683	+0.106
Meniscal	AUROC	0.7193	0.6247	+0.095
Meniscal	AUPRC	0.5147	0.3668	+0.148
Mean	AUPRC	0.5444	0.4175	+0.127

Single-task baselines used identical backbone, hyperparameters, augmentation, and cross-validation splits but were trained exclusively on the KL0/1 MRI-labeled subset without KL supervision from the full cohort.

## 4 Discussion

The observed prevalence gradient (cartilage damage rising from 21.6% in KL0 to 53.8% in KL1, and meniscal damage from 20.4% to 40.2%) indicates that radiographic grade, even within the early (KL0/1) stratum, carries incremental structural information, lending biological plausibility to the hypothesis that indirect radiographic features may encode some tissue-level risk signal.

This study demonstrates that a standard posteroanterior knee radiograph, processed by a multi-task deep learning model, can provide moderate discrimination of MRI-defined structural soft tissue pathology in knees with early radiographic osteoarthritis, while preserving high-quality KL grading across the full severity spectrum. The KL performance (mean QWK 0.8284) is consistent with published benchmarks from dedicated single-task KL architectures (Tiulpin and Saarakkala, 2020; Swiecicki et al., 2021; Brejnebøl et al., 2024; Zhao et al., 2024; Vaattovaara et al., 2025), indicating that incorporating MRI-supervised auxiliary heads did not substantially impair the primary grading capability. This is methodologically important, as it confirms that the multi-task architecture successfully shares early convolutional features across tasks without catastrophic interference.

The ablation analysis directly quantifies this benefit: single-task ConvNeXt-Base baselines trained only on the 2,561 KL0/1 labeled knees achieved AUROC of 0.662 for cartilage and 0.625 for meniscal endpoints, compared with 0.733 and 0.719 under the multi-task framework; absolute improvements of +0.071 and +0.095 in AUROC, and +0.106 and +0.148 in AUPRC. The magnitude of this improvement validates the core architectural rationale: KL supervision from the full 8,260-knee cohort develops richer radiograph representations than are achievable from the smaller MRI-labeled subset alone, and these representations transfer effectively to the MRI inference tasks.

The magnitude of MRI endpoint discrimination (AUROC 0.73 for cartilage damage, 0.72 for meniscal damage) should be interpreted in its proper methodological context. These values are substantially below those reported for MRI-input deep learning models applied to directly visualized soft tissue targets, where pooled AUC values exceeding 0.93 are documented for meniscal tears and cartilage phenotypes (Roblot et al., 2019; Fritz et al., 2020; Astuto et al., 2021; Rizk et al., 2021; Güngör et al., 2025). This gap is expected and does not undermine the clinical value of the present approach. Radiographs do not directly depict cartilage morphology or meniscal structure; any signal extracted for these endpoints must be derived from indirect radiographic correlates such as compartmental joint space, osteophyte geometry, alignment, and subchondral bone morphology. That AUROC values above 0.70 are achievable from radiograph-only inference in KL0/1 knees where the radiograph appears normal or near-normal to the reading clinician, is a meaningful finding, suggesting that the model is exploiting subtle but real radiographic features that carry structural correlates detectable by MRI.

The prevalence data reinforce the clinical relevance of the target population: nearly one third of KL0/1 knees harbored definite tibiofemoral cartilage damage (31.3%) and over one quarter had definite meniscal structural damage (26.4%) on MRI. These figures are consistent with epidemiological data from OAI and similar cohorts indicating that MRI-defined structural abnormalities frequently precede radiographic severity thresholds commonly used to trigger MRI referral (Chang et al., 2025; Chang et al., 2026). A triage model achieving AUROC ∼0.73 in this population translates to a meaningful enrichment of the pool referred for MRI relative to unguided radiographic staging, particularly when operating thresholds are tuned toward sensitivity in a triage paradigm. At the validation-derived best-F1 threshold, sensitivity of approximately 0.74 for cartilage damage and 0.68 for meniscal damage was achievable, accompanied by specificity around 0.63-0.65, which is operationally useful for a first-line filter task.

The predicted-KL gating sensitivity analysis is important for implementation realism. Deploying MRI inference heads in an automated pipeline requires upstream KL prediction to determine early-disease routing. The observed mean AUPRC reduction of 0.0321 under predicted-KL gating quantifies this cascade cost, while paired testing showed non-significant fold-level differences (*p* = 0.375). The conformal analysis adds a practical reliability layer: uncertain cases can be flagged through multi-label prediction sets with controlled coverage rather than over-interpreted as definitive positives or negatives.

Several prior studies have explored AI-based soft tissue inference from knee radiographs but have addressed narrower tasks or applied different methodological frameworks. Chen et al. used deep learning on knee radiographs to detect bucket-handle meniscal tears, a structurally extreme and often radiographically visible variant that differs from the early morphological MOAKS-based endpoints targeted here (Chen et al., 2026). No prior study, to our knowledge, has simultaneously trained full-spectrum KL grading and early-KL-specific MOAKS-grounded cartilage and meniscal endpoints within a single architecture under strict leakage-controlled cross-validation. The methodological contributions of the present work are therefore: (1) explicit MRI-endpoint supervision restricted to the intended early-disease subgroup; (2) subject-level cross-validation preventing patient-level leakage; (3) pre-specified checkpoint selection; and (4) quantification of cascade loss from upstream KL prediction uncertainty.

An important clinical caveat must be emphasized. The model provides a probabilistic radiograph-derived signal intended to support, but not replace, clinical judgment in MRI referral decisions. The decision to request MRI must remain grounded in the individual patient’s symptoms, functional limitations, age, body mass index, prior treatment history, and patient preferences — consistent with the principle of treating the patient rather than the imaging finding. A high model-predicted probability does not constitute an automatic MRI indication, just as a low probability does not preclude MRI when the clinical picture warrants it. The model is intended as a population-level risk enrichment tool: flagging radiographically mild knees where the prior probability of structural soft tissue damage is elevated, making MRI referral decisions more informed rather than making them for the clinician. This distinction is critical to responsible implementation.

This study has several limitations. First, it is a retrospective single-cohort development study (OAI), and all primary performance estimates are from internal cross-validation. External validation in an independent KL0/1 cohort remains necessary before clinical translation. Second, MRI endpoints are knee-level non-localizing composites, which limits compartment-specific interpretability. Third, approximately 46% of KL0/1 knees lacked complete MRI endpoint labels; although included-versus-excluded knees showed small observed differences in age and KL stratum, residual selection bias from unmeasured factors cannot be excluded. Fourth, MOAKS-based MRI scoring is an accepted non-invasive reference but not perfect ground truth relative to arthroscopy. Fifth, saliency maps are associative rather than causal and should be interpreted as supportive evidence only (**Supplementary Figure 5**).

Future work should prioritize multi-center external validation in independent KL0/1 cohorts with MRI reference labels, region-level endpoint modeling, and prospective workflow-impact evaluation. In this revision, exploratory leakage-safe fusion with available covariates (age and KL) improved MRI endpoint discrimination relative to imaging-only probabilities, supporting planned multimodal extensions when broader covariates (e.g., BMI, surgery history, symptom severity) are harmonized.

## 5 Conclusions

A leakage-controlled, reproducible multi-task deep learning model trained on standard knee radiographs achieved strong Kellgren-Lawrence grading agreement and moderate discrimination of MRI-defined tibiofemoral cartilage and meniscal morphology damage in knees with early radiographic osteoarthritis. The framework demonstrates that clinically meaningful risk stratification for MRI-level soft tissue pathology may be extractable from first-line radiographic evaluation, supporting a potential role as an assistive triage signal to identify patients who may benefit from expedited MRI assessment. The clinical decision to request MRI must remain anchored in comprehensive patient evaluation; the model output should be regarded as one probabilistic input among many rather than a standalone referral criterion.

## Data Availability

The raw data supporting the conclusions of this article will be made available by the authors, upon reasonable requests to the corresponding author.
