# Supplementary material for: Standard knee radiographs enable deep learning inference of MRI-defined cartilage and meniscal damage in early knee osteoarthritis: a study using the osteoarthritis initiative database
Source: Front Physiol. 2026 Jun 10;17:1858407. doi: 10.3389/fphys.2026.1858407 (PMC13290120; doi:10.3389/fphys.2026.1858407)
Supplement: Supplementary file 1 [file DataSheet1.docx]

Supplementary Material


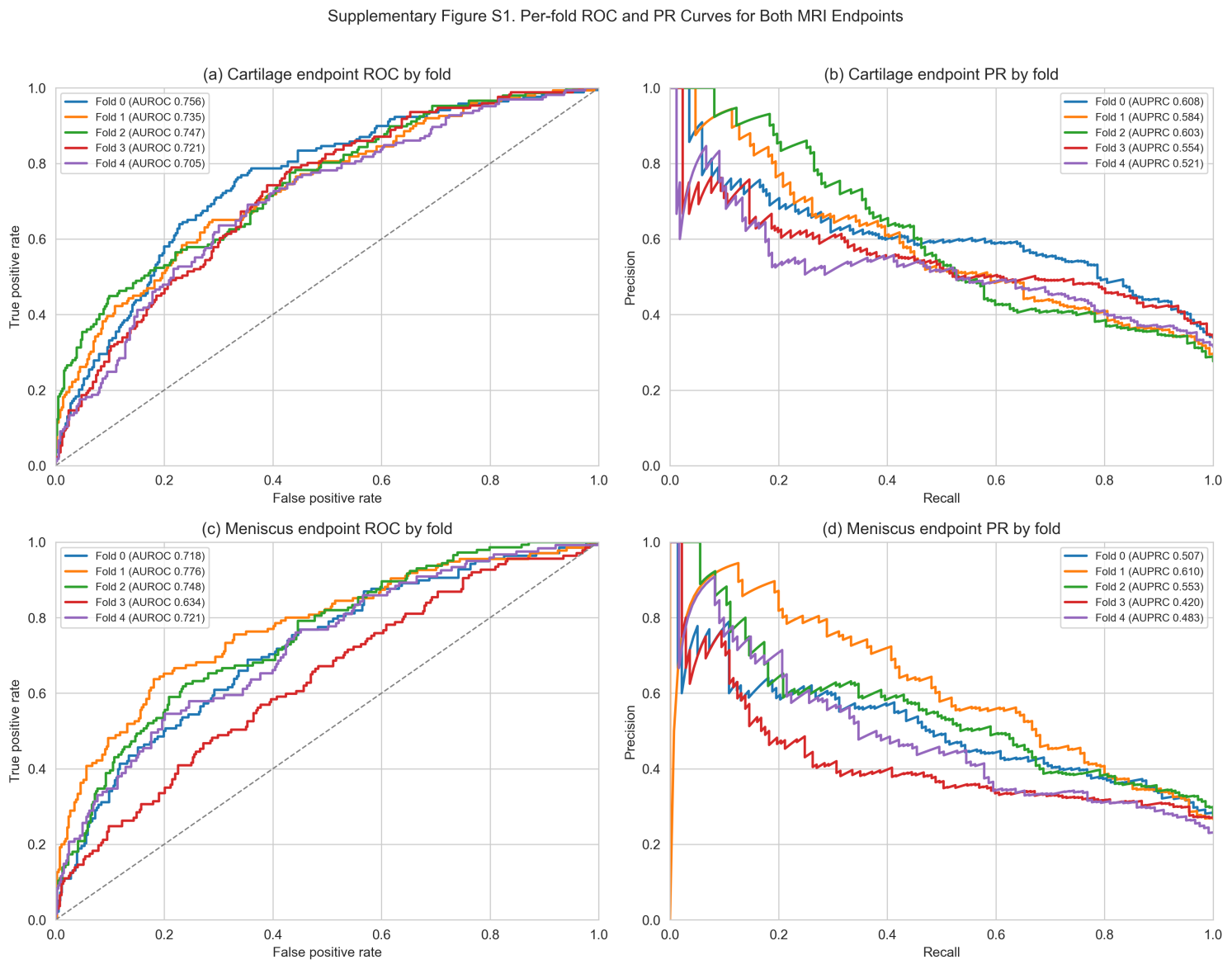


**Figure S1.** Per-fold ROC and PR curves for both MRI endpoints. (A) ROC curves across folds 0-4 for the tibiofemoral cartilage endpoint. (B) PR curves across folds 0-4 for the tibiofemoral cartilage endpoint. (C) ROC curves across folds 0-4 for the meniscal morphology endpoint. (D) PR curves across folds 0-4 for the meniscal morphology endpoint. Legends report per-fold AUROC/AUPRC.


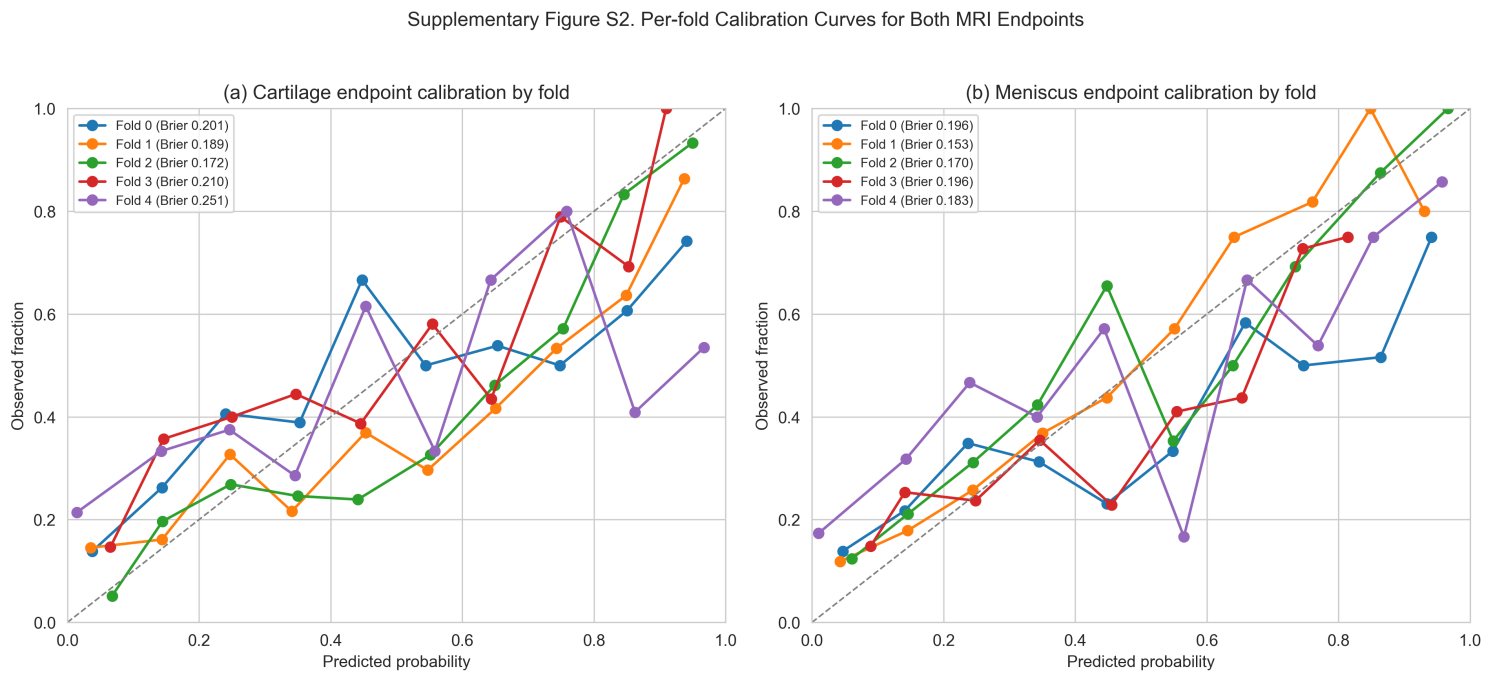


**Figure S2.** Per-fold calibration curves for both MRI endpoints. (A) Reliability curves for the tibiofemoral cartilage endpoint across folds 0-4. (B) Reliability curves for the meniscal morphology endpoint across folds 0-4. The diagonal indicates perfect calibration; legends report per-fold Brier scores.


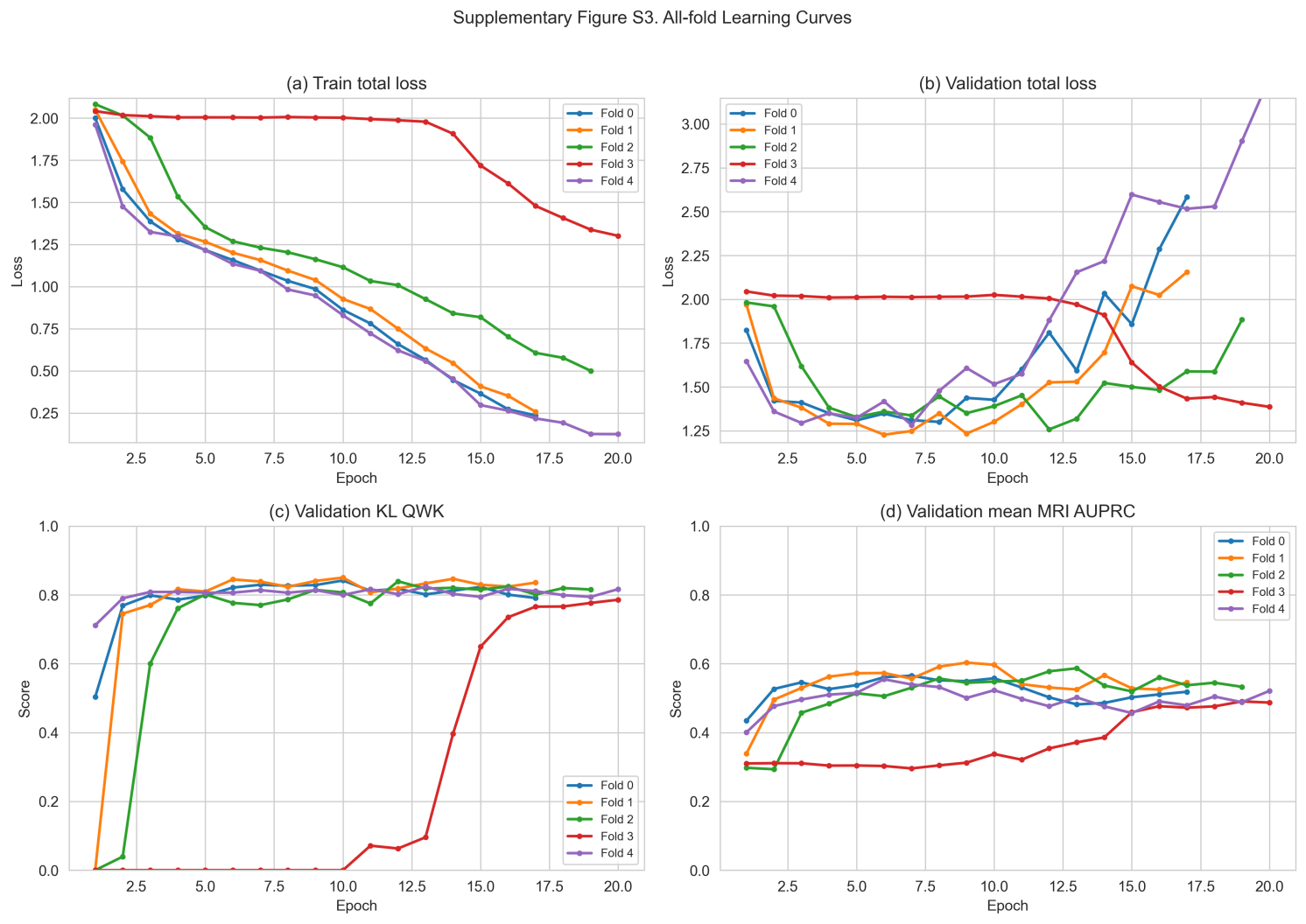


**Figure S3.** All-fold learning curves. (A) Train total loss. (B) Validation total loss. (C) Validation KL QWK. (D) Validation mean MRI AUPRC across epochs for folds 0-4.


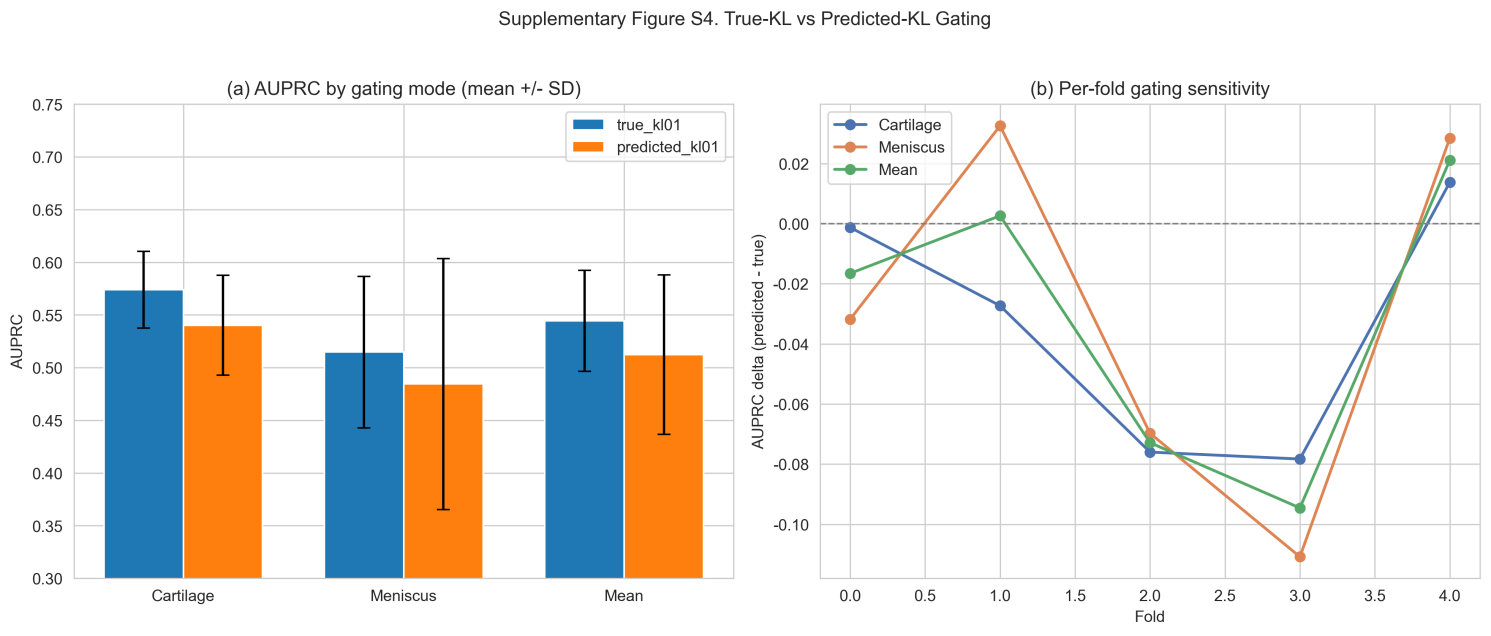


**Figure S4.** True-KL versus predicted-KL gating sensitivity analysis. (A) Mean +/- SD AUPRC under true-KL gating versus predicted-KL gating for cartilage endpoint, meniscus endpoint, and their mean. (B) Per-fold AUPRC delta (predicted-gating minus true-gating), showing performance change attributable to deployment-style predicted-KL gating.


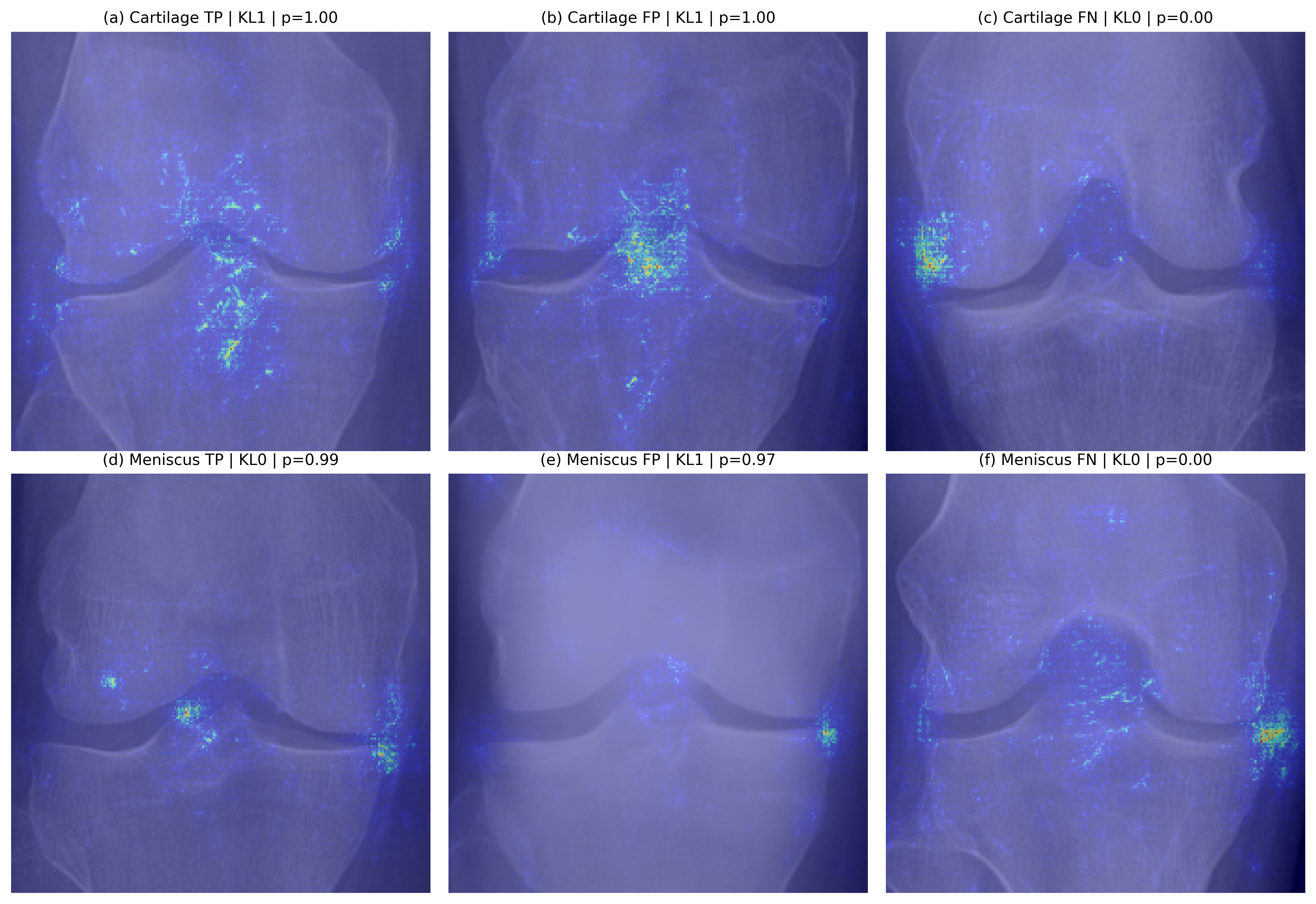


**Figure S5.** SmoothGrad saliency overlays for MRI-endpoint predictions in representative held-out knees. Panels include representative true-positive, false-positive, and false-negative examples for cartilage and meniscal endpoints; warmer colors indicate regions with greater contribution to endpoint logits.

**Supplementary Tables**

**Table S1.** Missing-label bias comparison between included and excluded KL0/1 knees.

| **Variable** | **Included** | **Excluded** | **SMD** |
| --- | --- | --- | --- |
| N knees | 2561 | 2187 |  |
| Age, mean (SD) | 59.50 (8.96) | 60.15 (9.35) | -0.071 |
| KL1, n (%) | 772 (30.1%) | 723 (33.1%) | -0.063 |

**Table S2.** True-KL versus predicted-KL gating paired comparison (AUPRC).

| **Metric** | **True-KL gated mean** | **Pred-KL gated mean** | **Difference (Pred-True)** | **95% CI of difference** | ***P* value** |
| --- | --- | --- | --- | --- | --- |
| Mean MRI AUPRC | 0.544 | 0.512 | -0.032 | -0.071 to 0.006 | 0.375 |
| Cartilage AUPRC | 0.574 | 0.54 | -0.034 | -0.067 to -0.000 | 0.188 |
| Meniscus AUPRC | 0.515 | 0.484 | -0.03 | -0.079 to 0.018 | 0.375 |

**Table S3.** Conformal uncertainty summary across endpoints and alpha levels.

| **Endpoint** | **Alpha** | **Target coverage** | **Empirical coverage** | **Singleton rate** | **Average set size** |
| --- | --- | --- | --- | --- | --- |
| Cartilage | 0.1 | 0.9 | 0.9 | 0.38 | 1.62 |
| Cartilage | 0.2 | 0.8 | 0.8 | 0.669 | 1.331 |
| Meniscus | 0.1 | 0.9 | 0.892 | 0.349 | 1.651 |
| Meniscus | 0.2 | 0.8 | 0.796 | 0.652 | 1.348 |

**Table S4.** Subgroup performance summary (overall, KL strata, and age strata).

| **Subgroup type** | **Subgroup** | **Endpoint** | **N mean/fold** | **Prevalence** | **AUROC** | **AUPRC** |
| --- | --- | --- | --- | --- | --- | --- |
| Age subgroup | 55-64 | Cartilage | 170.2 | 0.327 | 0.703 | 0.545 |
| Age subgroup | 55-64 | Meniscus | 170.2 | 0.275 | 0.718 | 0.509 |
| Age subgroup | <55 | Cartilage | 183.6 | 0.218 | 0.755 | 0.491 |
| Age subgroup | <55 | Meniscus | 183.6 | 0.171 | 0.722 | 0.433 |
| Age subgroup | >=65 | Cartilage | 158.4 | 0.41 | 0.717 | 0.67 |
| Age subgroup | >=65 | Meniscus | 158.4 | 0.359 | 0.695 | 0.592 |
| KL subgroup | KL0 | Cartilage | 357.8 | 0.216 | 0.701 | 0.403 |
| KL subgroup | KL0 | Meniscus | 357.8 | 0.205 | 0.693 | 0.412 |
| KL subgroup | KL1 | Cartilage | 154.4 | 0.536 | 0.679 | 0.729 |
| KL subgroup | KL1 | Meniscus | 154.4 | 0.403 | 0.697 | 0.628 |
| Overall | All | Cartilage | 512.2 | 0.313 | 0.733 | 0.574 |
| Overall | All | Meniscus | 512.2 | 0.264 | 0.719 | 0.515 |

**Table S5.** Exploratory covariate fusion analysis (age and KL).

| **Endpoint** | **Model** | **AUROC** | **AUPRC** |
| --- | --- | --- | --- |
| Cartilage | Image model only | 0.733 | 0.574 |
| Cartilage | Age+KL only | 0.712 | 0.537 |
| Cartilage | Image+Age+KL fusion | 0.758 | 0.606 |
| Meniscus | Image model only | 0.719 | 0.515 |
| Meniscus | Age+KL only | 0.674 | 0.416 |
| Meniscus | Image+Age+KL fusion | 0.733 | 0.526 |

**Table S6.** Operating-point summary at threshold 0.5 and validation-derived best-F1 threshold.

| **Endpoint** | **Sensitivity @0.5** | **Specificity @0.5** | **Sensitivity @best-F1** | **Specificity @best-F1** | **Best-F1 threshold (mean)** |
| --- | --- | --- | --- | --- | --- |
| Cartilage | 0.435 | 0.842 | 0.739 | 0.626 | 0.185 |
| Meniscus | 0.301 | 0.919 | 0.683 | 0.652 | 0.164 |
